# Behavioral changes in response to sound exposure and no spatial avoidance of noisy conditions in captive zebrafish

**DOI:** 10.3389/fnbeh.2015.00028

**Published:** 2015-02-17

**Authors:** Yik Yaw Neo, Lisa Parie, Frederique Bakker, Peter Snelderwaard, Christian Tudorache, Marcel Schaaf, Hans Slabbekoorn

**Affiliations:** ^1^Behavioral Biology, Institute of Biology (IBL), Leiden UniversityLeiden, Netherlands; ^2^Naturalis Biodiversity CenterLeiden, Netherlands

**Keywords:** decision-making, noise impact, captive fish behavior, preference test, sound exposure

## Abstract

Auditory sensitivity in fish serves various important functions, but also makes fish susceptible to noise pollution. Human-generated sounds may affect behavioral patterns of fish, both in natural conditions and in captivity. Fish are often kept for consumption in aquaculture, on display in zoos and hobby aquaria, and for medical sciences in research facilities, but little is known about the impact of ambient sounds in fish tanks. In this study, we conducted two indoor exposure experiments with zebrafish (*Danio rerio*). The first experiment demonstrated that exposure to moderate sound levels (112 dB re 1 μPa) can affect the swimming behavior of fish by changing group cohesion, swimming speed and swimming height. Effects were brief for both continuous and intermittent noise treatments. In the second experiment, fish could influence exposure to higher sound levels by swimming freely between an artificially noisy fish tank (120–140 dB re 1 μPa) and another with ambient noise levels (89 dB re 1 μPa). Despite initial startle responses, and a brief period in which many individuals in the noisy tank dived down to the bottom, there was no spatial avoidance or noise-dependent tank preference at all. The frequent exchange rate of about 60 fish passages per hour between tanks was not affected by continuous or intermittent exposures. In conclusion, small groups of captive zebrafish were able to detect sounds already at relatively low sound levels and adjust their behavior to it. Relatively high sound levels were at least at the on-set disturbing, but did not lead to spatial avoidance. Further research is needed to show whether zebrafish are not able to avoid noisy areas or just not bothered. Quantitatively, these data are not directly applicable to other fish species or other fish tanks, but they do indicate that sound exposure may affect fish behavior in any captive condition.

## Introduction

The world is becoming increasingly noisy due to all sorts of motorized road, rail and air traffic and a huge variety of industrial and recreational activities. Such noise pollution originating from anthropogenic sources is considered a critical public health problem by the World Health Organization (WHO, 2011) and has potential for detrimental effects of both auditory and non-auditory nature (Miedema, 2007; Szalma and Hancock, 2011; Basner et al., 2014). Extreme over-exposure may cause temporary or permanent threshold shifts for hearing, while more moderate sound levels may cause disturbance, masking, or distraction, all having an impact on decision-making processes in the brain (e.g., Banbury et al., 2001; Starcke and Brand, 2012; van Gaal et al., 2012). Also animals can be affected in these ways by elevated environmental noise levels of anthropogenic origin in air (Slabbekoorn and Ripmeester, 2008; Kight and Swaddle, 2011; Francis and Barber, 2013) and in water (Richardson et al., 1998; Southall et al., 2007; Slabbekoorn et al., 2010; Radford et al., 2014).

Fish are known to depend on sounds for a variety of functions that are critical for survival and reproduction (Fay and Popper, 2000; Ladich, 2008; Fay, 2009) and may therefore be negatively affected by noise pollution (Popper et al., 2003; Popper and Hastings, 2009; Slabbekoorn et al., 2010; Radford et al., 2014). Although the available data are often anecdotal, badly replicated or concerning artificial conditions, it has been shown that experimental noise exposure can lead to increased heart rate or elevated cortisol levels (e.g., Santulli et al., 1999; Wysocki et al., 2006; Graham and Cooke, 2008). Also behavioral changes are reported such as interruption of courtship and spawning activity (Boussard, 1981; Slabbekoorn et al., 2012) and alteration of territorial behavior (Sebastianutto et al., 2011). Several fish species appear to have a tendency to dive into deeper water in response to vessel noise (Gerlotto and Fréon, 1992; Draštík and Kubečka, 2005; Sarà et al., 2007). Spatial avoidance reactions in the horizontal plane are reported in the context of air guns and seismic surveys (Engås et al., 1996; Hirst and Rodhouse, 2000; Peña et al., 2013). However, other studies only report brief startle responses (see e.g., Wardle et al., 2001; Løkkeborg et al., 2012) and again the data available are very limited and do not allow any inference yet about presence or absence of negative effects (Slabbekoorn, 2012).

Noise impact data are challenging to collect on free-ranging fish in the field, but fish are also exposed to sound in captivity, e.g., when kept for consumption in aquaculture, on display in zoos and hobby aquaria, and for medical sciences in research facilities (Bart et al., 2001; Davidson et al., 2007; Wysocki et al., 2007; Craven et al., 2009). Ambient sounds in these places can include continuous hums and repetitive clicks or sound bursts, but also unpredictable switches in noise conditions (from maintenance systems or cleaning and construction machinery) or sudden and loud peaks (from slamming doors, dropping heavy objects or knocking on tank walls). Sounds typically travel well through concrete walls and floors, metal and wooden tables, into glass or plastic fish tanks. Such sounds differ from each other in terms of their frequency ranges, amplitudes and temporal patterns, which are parameters that are likely to affect their masking, deterrent and interruptive impact (Vasconcelos et al., 2007; Codarin et al., 2009; Neo et al., 2014).

In addition to the above mentioned exposure parameters, it is important to realize that indoor fish enclosures typically have very complex sound fields for a variety of reasons. These reasons include: primary and secondary sound source variety, near field conditions, wavelengths of relevant frequencies typically exceeding tank measures, reflections and refractions, and sound pressure release at water-air interfaces (Parvulescu, 1967; Akamatsu et al., 2002). Furthermore, captive fish can be more or less domesticated and behavioral response opportunities are obviously restricted by tank dimensions and affected by hiding options and the composition and density of the tank community (e.g., Calisi and Bentley, 2009; Benhaim et al., 2013; Slabbekoorn, in press). Therefore, response patterns under these captive conditions do not necessarily reflect response patterns of wild-ranging fish to sounds in natural conditions and remain largely unexplored (but see Kastelein et al., 2008; Purser and Radford, 2011; Neo et al., 2014; Voellmy et al., 2014).

The zebrafish (*Danio rerio*) is a common freshwater aquarium species with growing popularity in a variety of scientific disciplines, including research into vertebrate hearing (Higgs et al., 2002, 2003; Whitfield, 2002; Zeddies and Fay, 2005) as well as pharmacological investigations into stress and anxiety (Egan et al., 2009; Champagne et al., 2010; Gerlai, 2010; Steenbergen et al., 2011). Zebrafish are not known to produce sounds for communication, but have excellent hearing abilities owing to the presence of the Weberian ossicles between the swim bladder and the inner ears. They are able to hear sounds best between 300 and 2000 Hz and have a sensitivity peak around 800 Hz (Higgs et al., 2002). Zebrafish are very suitable for noise impact studies on freshwater fish because of the ease of maintenance, and their shoaling and continuous swimming behavior, which allows detailed analyses of their response behavior to external stiumuli through quantification of their swimming and spatial behavior (Egan et al., 2009; Cachat et al., 2010; Gerlai, 2010; Gaikwad et al., 2011). We recently also confirmed stressor-related behavioral and physiological covariation for both adult and larval zebrafish of different coping styles (Tudorache et al., 2013, 2015).

In this study, we explored the nature of the behavioral response of small groups of captive zebrafish to exposure with moderate sound levels, i.e., well below the expected threshold for physical harm, but likely to be within the audible range (Hawkins and Popper, 2012). We conducted two complementary experiments exploring variation in effect from continuous and intermittent noise treatments. In experiment I, we investigated the potential for sound to affect group cohesion, swimming speed and swimming height, using noise treatments of 112 dB re 1 μPa in a single tank without escape possibility (Acoustic exposure test). We aimed at finding an answer to the following question: Does moderate sound exposure induce behavioral changes in captive zebrafish? In experiment II, we investigated the potential for sound to affect spatial distribution in terms of the tendency to enter and leave a noisy area when a quiet alternative is available, using noise treatments of up to 140 dB re 1 μPa in a double-tank system with escape possibility (Auditory preference test). The question that we aimed to answer here was: Do captive zebrafish spatially avoid moderately loud sound conditions that are disturbing at least at their on-set.

## Methods

### Experiment I (acoustic exposure test)

#### Animal maintenance

Around 200 zebrafish individuals of mixed sex were housed together in a long tank (200 × 40 × 50 cm) containing plastic aquatic plants and connected to a water recirculation system. The fish were purchased from a commercial stock as juveniles and were approximately 5–6 months old at the time of the experiments. Fish were fed commercial feed every other day and kept in a 12:12 dark:light cycle. Water temperature was maintained at 25 ± 1°C. All experiments were performed in accordance with the Netherlands Experiments on Animals Act (DEC approval no: 10069) that serves as the implementation of the Directive 86/609/EEC by the Council of the European Communities regarding the protection of animals used for experimental and other scientific purposes (1986). After the study, the animals were kept for breeding and re-use in subsequent experiments.

#### Treatment preparation

**Figure 1:**
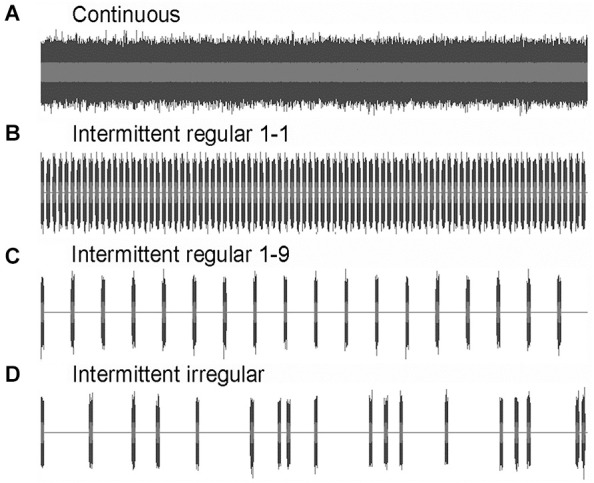
**Amplitude waves of the four noise treatments used in the exposure experiments I and II: (A) continuous; (B) intermittent regular: 1 s + 1 s; (C) intermittent regular: 1 s + 9 s; and (D) intermittent irregular: 1 s + 1–17 s**.

Four noise treatments were used in the experiments: continuous, intermittent regular 1-1, intermittent regular 1-9 and intermittent irregular (Figure 1). Sound samples were created in Audacity 1.2.6 software, using full-spectrum white noise, band-pass filtered between 500 and 1500 Hz. The details of the sound spectrum at the fish will depend on the output characteristics of the speakers and the transmission into the tank, but this broad-band and band-passed sound stimulus guaranteed that the elevation above ambient in measured sound pressure level (SPL) reflects an elevation in the relevant hearing range of the zebrafish (300–2000 Hz, Higgs et al., 2002). Intermittent noise treatments consisted of playback of 1 s pulses of the same spectrum, but were different from each other in terms of the length of the silent intervals. Regular 1-1 noise consisted of 1 s pulses interspersed with 1 s silent intervals (1 s + 1 s); Regular 1-9 noise consisted of 1 s pulses interspersed with 9 s silent intervals (1 s + 9 s); Irregular noise consisted of 1 s pulse interspersed with 1 to 17 s silent intervals (1 s + 1–17 s), leading to a mean silent interval of 9 s comparable to regular 1-9. The silent intervals in subsequent exposures of the irregular noise were determined randomly with an online random number generator.^1^

#### Experimental set-up

**Figure 2:**
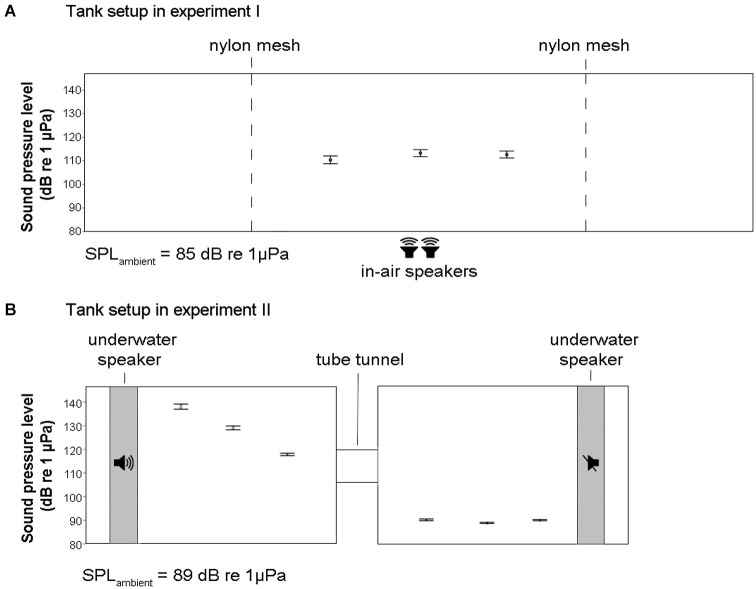
**Schematic diagrams of the tanks for the experimental set-up of experiment I and II with SPL measurements across several longitudinal positions in the tanks (±SE)**. At each position, nine measurements across the width and depth of the tanks were taken and averaged. **(A)** In experiment I, an enclosed area of a long tank was used together with two in-air speakers placed on the ground under the tank table (1 m height). There was an increase in SPL of 27 dB re 1 μPa in the tank during noise exposure. **(B)** In experiment II, a double-tank system was used together with two underwater speakers. There was an increase in SPL of about 40 dB re 1 μPa in the tank with active speaker during noise exposure.

The experimental trials were conducted in a single glass tank (200 × 60 × 50 cm), which stood on four metal legs 80 cm above the ground (Figure 2A). The experimental area was reduced to half of the tank length (100 × 60 × 50 cm) using two dividers made of black PVC plate frames and nylon mesh (water level was kept constant throughout the experiments at 48 cm from the bottom). Black background and 2 cm of black sand on the bottom were used to increase the contrast of the fish for the digital tracking (one frame per second) with Ethovision XT 6.1.326 (Noldus, The Netherlands) based on the continuous side-view camera recordings (individual tracking could get mixed up when two individuals cross each other’s 2D-pathways, but this does not affect measurements on the target group averages). The 2D-assessment of speed and distance among individual fish is an underestimate, not taking the space from front to back fully into account, but this is consistent among treatments and does not undermine the validity of our test of exposure impact or treatment effect. The water was again connected to a recirculation system and the temperature was kept at 25 ± 1°C. During the test trials, the water recirculation system was switched off in order to reduce the potential impact of any associated sound or water flow (the difference in ambient SPL was about 3 dB re 1 μPa and the switch-off, more than 1 h before the on-set of the first sound exposure treatment, did not lead to obvious changes in fish behavior).

In each trial, five fish from the stock population were placed in the tank prior to the experiments and allowed at least 16 h of acclimatization for morning trials (overnight) and at least 3 h of acclimatization for afternoon trials. We tested for an effect of acclimatization difference and morning vs. afternoon trials and found no significant impact on the results. It is good to realize that baseline behavior as well as response patterns may be affected by having at least 3 h of acclimatization, but also that they are specific for tank size, temperature and light conditions as well as for species and group size, age and composition. Even within species, domestication effects and strain differences in behavior may exist (e.g., Huntingford, 2004; Mahabir et al., 2013). We therefore used a single species and animals from the same strain and domesticated background and investigated exposure and treatment effects by comparing repeated measures of the same group and used a replicate set of same-sized groups in the same conditions to reduce variation due to factors beyond the interest of the current paper.

Each group was only exposed to one treatment in each trial and 40 exposure trials were conducted in total (40 × 5 = 200 experimental fish). The noise treatments were played back from two in-air speakers (CB4500, Blaupunkt, Germany) placed below the tank with a solid state audio recorder (PMD670, Marantz, Japan) connected to an amplifier (AX-R561, JVC, Japan). The whole trial (45 min) was recorded with a video recorder (GZ-MG505E, JVC, Japan) and included 15 min noise exposure and 15 min before and 15 min after exposure. The experimenter controlled the video from behind a curtain and the early, single video switch-on moment for the whole trial prevented any impact on zebrafish behavior around noise on-set and off-set moments of noise exposure. Nine to eleven replicates were used for each treatment (one mistake in stimulus selection reduced sample size for one treatment and increased it for another). The order of all treatment trials was randomized to avoid the effect of treatment being confounded with date or time in the sequence of trials.

#### Sound field conditions

We assessed SPL from the recordings with a hydrophone (HTI 96 min, High Tech, US) connected to a solid state audio recorder (PMD620, Marantz, Japan) at three different longitudinal positions in the tank, where we took nine measurements each, sampling variation with height and width (3 × 3 positions) during playback of continuous noise (wav format, 44.1 kHz sampling rate). The SPLs were calculated as root mean square (RMS) in Matlab (7.0.1) using a script calibrated for the recording set within the 300–2000 Hz frequency range (matching the auditory sensitivity of zebrafish). The SPL at playback was 112 dB re 1 μPa on average (with minor spatial variation and the lowest measurement level in the center of the tank) which was considerably higher than the “silent” control which ambient levels of 85 dB re 1 μPa (Figure 2A). Zebrafish sensitivity to sound is likely dominated by the sound pressure component, but they also are sensitive to particle motion especially in the low frequencies (Higgs et al., 2002, 2003; Popper and Fay, 2011; Bretschneider et al., 2013). Using in air speakers to ensonify the tank makes sound enter from all sides (table and tank walls serve as secondary sound source) and causes sound pressure to vary little throughout the fish tank, while particle motion is much more variable in all directions (inherent to near-field conditions and small tank size relative to wave length) providing a highly complex sound field to the fish and the investigator (Parvulescu, 1967). However, it is important for the current experiments that both average sound pressure and average particle motion are elevated significantly to allow exploration of the nature of sound induced changes in captive zebrafish and for testing variation of impact for different temporal patterns of the same exposure level (c.f. Voellmy et al., 2014).

#### Behavioral observations and analyses

Video recordings were analyzed with Ethovision software, measuring group cohesion (average distance between individuals of all possible pairs), swimming speed (averaged for all individuals as measured on the screen in two dimensions) and the duration of the time spent at different depths (four vertical sections). The latter measurement was reduced to a single assessment of time present within the upper layer closest to the surface as this yielded a proper quantification of the observation that fish were often seen to move upward right after noise exposure on-set. Measurements were averaged over each one-minute period for the whole trial (45 min). Startle responses are expected especially at the on-set of sound exposure and are defined as a sudden contraction of the body in a typical c-shape, followed by a distinct swimming burst, often at a shifted angle compared to pre-startle swimming speed and direction (Kastelein et al., 2008; Stewart et al., 2012). In this experiment, startle responses are reflected in elevated speed in the first minute of exposure.

#### Statistics

The data were analyzed with SPSS 16.0. To find out if swimming behavior changed across the trial periods, repeated measures ANOVAs were performed, with “treatment” (continuous, regular 1-1, regular 1-9, irregular) as a between-subjects factor and “period” (before, during1, during2, after) as a within-subjects factor. In addition, repeated measures ANOVAs were also conducted for each treatment separately to test for significant changes in each treatment. Since most changes were seen during the first few minutes after noise exposure, time was grouped into four 5 min bins: the last 5 min before noise exposure (“before”), the first 5 min during noise exposure (“during1”), the last 5 min during noise exposure (“during2”), and the first 5 min after noise exposure (“after”). We used Huynh-Feldt corrections when sphericity could not be assumed in a test. We conducted *post hoc* Bonferroni tests when a factor was significant.

### Experiment II (auditory preference test)

#### Treatment preparation

We used a similar approach to experiment I, using noise exposure stimuli of filtered white noise (now band-passed between 300–2000 Hz, still aiming for elevation of ambient levels within the relevant hearing range of zebrafish) varying in temporal pattern. However, due to practical constraints, we omitted the intermittent regular 1-1 treatment in experiment II, consequently testing only three instead of four temporal treatments.

#### Experimental set-up

The experimental trials were conducted in a double-tank system, with two tanks (75 × 50 × 50 cm each) linked together with a tube tunnel (length: 35 cm, diameter: 12.5 cm), allowing fish to swim between the tanks freely (Figure 2B). The bottom of each tank was insulated acoustically with a layer of Styrofoam, a layer of glass fiber and four rubber pads before the tanks were placed on trolleys with rubber wheels, to minimize sound transmission through the floor. We now used two underwater speakers (DRS-8, Oceanears, US), housed in Styrofoam and installed at the end of both tanks opposite to the tube entrance (in air speakers like in experiment 1 would not induce the required sound level differences between tanks). A layer of Styrofoam was also placed in the tanks at the tube tunnel end to cover the protruding parts of the tube tunnel. Sand was used to fill up the gaps between the Styrofoam and the tank floor on both sides of each tank to prevent fish from swimming into the restricted areas. The system was connected to a water recirculation system with water flowing in from behind the speaker in one tank and out from the other tank (water level was kept constant again throughout the experiments at 48 cm from the bottom). During the treatments, the water recirculation system was switched off (mainly to avoid an impact of water flow through the connecting tube on fish exchange between tanks), while the tank sides for noise exposure were alternated to avoid an impact of side preferences (Butman and Grassle, 1992) or other spatial factors.

In each trial, six fish from the stock population (not used in experiment I and naïve to experimental sound exposure) were placed in the experimental tanks (three on each side) prior to the experiment and allowed at least 16 h of acclimatization (overnight). The noise treatment files were played back from the speakers with a solid state audio recorder (PMD620, Marantz, Japan) connected to an amplifier (AX-R562, JVC, Japan). Both speakers were on during experiments but only one speaker was playing a noise treatment file (active speaker vs. quiet speaker). The noise exposure lasted for 1 h, which is much longer than in the first experiment to allow more long-term spatial effects of noise avoidance. A whole trial (3 h), including 1 h before, 1 h during and 1 h after noise exposure, was recorded with a video recorder (GZ-MG505E, JVC, Japan). Eight replicates were used for each treatment; the treatment (active speaker) was in the left tank of the system for four of the replicates and in the right tank for the other four. Each group was only exposed to one of the three treatments and consequently exposure trials on 24 groups were conducted in total (8 groups × 3 treatments × 6 individuals = 144 experimental fish).

#### Sound field conditions

SPLs (RMS) were measured at 27 locations (in 3 × 3 × 3 matrix) of the each tank. As shown in Figure 2B, the SPL of the “silent” tank was on average 89 dB re 1 μPa and we were able to generate a difference in SPL between the two tanks of up to 50 dB re 1 μPa. Moreover, within the treatment tank, there was a gradient in SPL ranging from around 120 dB re 1 μPa close to the tunnel to around 140 dB re 1 μPa close to the speaker. Again, particle motion conditions are likely to be highly variable in all directions and are expected to be very complex. However, important for the set-up is that the tank difference in average levels of particle motion represent a similar contrast as SPLs in being much higher in the noisy tank than in the quiet tank. Fish switching tanks during the exposure period experience a distinct and synchronous transition from ambient to high levels (or vice versa) of both sound pressure and particle motion.

#### Behavioral observations and analyses

Video recordings were analyzed manually after the experiments, with treatment type “blind to the observer” (i.e., without the audio component of the recording). The focus here was not on detailed and short-term responses at the on-set of exposure, which are better described by the digital tracking method used for the first experiment, but on possibly more long-term effects of spatial avoidance after an initial exposure period that was expected to induce more startle responses due to the considerably higher sound levels compared to the first experiment. The first minute of the pre-exposure period of the videos was discarded to eliminate the influence of the experimenter leaving the room. Fish number in each tank was recorded at the start of each minute for the whole session (3 h) and the number of crossings between tanks was counted within every minute. If a fish was in the tube tunnel between the tanks during quantification, it was counted as being in the tank in which it had been seen last. The number of crossings is the sum of all crossings between the tanks for all six fish in the system.

#### Statistics

To find out if the number of crossings and the number of fish varied before and during treatments, the data before noise exposure from all treatments were grouped into a “control”. Subsequently, we conducted one-way ANOVAs with the number of crossings/the number of fish as dependent variable and “treatment” (pre-exposure control, continuous, regular 1-9, irregular) as independent variables. A general repeated measures ANOVA was used first (and reported), but we added the unconventional test with pre-exposure period grouped as control because in one of the treatments the number of fish in the treatment tank before noise exposure deviated from the expected value “3” by chance (inducing an obvious and unwanted type I error).

## Results

### Experiment I (acoustic exposure test)

**Figure 3:**
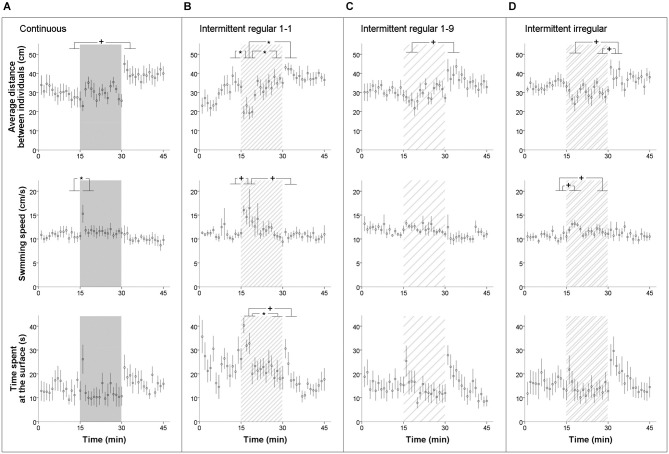
**Behavioral response measures on groups of five zebrafish in the acoustic exposure test of experiment I: average distance between individuals, swimming speed and time spent in upper quarter of the tank (±SE) across time in (A) continuous, (B) intermittent regular 1-1, (C) intermittent regular 1-9 and (D) intermittent irregular noise treatment**. The time was divided into four period bins for formal statistical analyses: the last 5 min before noise exposure (“before”), the first 5 min during noise exposure (“during1”), the last 5 min during noise exposure (“during2”), and the first 5 min after noise exposure (“after”). Horizontal bars at the significance indicators reflect the range of five one-minute samples in a five-minute bin as used for testing. *P* < 0.05*, *P* < 0.1^+^.

The groups of five zebrafish typically swam in loose shoals through the fish tank, often on their own and often together with one or two others within a distance of one or two body lengths. When exposed to moderate sound levels of 112 dB re 1 μPa, their behavior clearly altered, with startle responses at the on-set, decreased inter-individual distances and brief increases in swimming speed (we often saw one individual speeding towards another to slow down an split up again afterwards), while they swam more often in the highest water layer close to the surface (speeding and approaching often concerned upward moves to individuals that were relatively high in the water column). We never saw fish diving down the water column, staying close to the bottom or freezing. We found significant noise-dependent variation in all three behaviors across the experimental periods (Figure 3).

Firstly, when all treatments were analyzed together to characterize and test the overall impact of noise, group cohesion had a significant effect across periods (repeated measures ANOVA: *F*_(2.3,79.9)_ = 23.564, *P* < 0.001). Bonferroni-corrected *post hoc* tests revealed a significant decrease in the average distance at the onset of noise exposure (“during1” compared to the other three periods, all *P*s < 0.001) and a significant increase right after the noise exposure (“after” compared to the other three periods, all* P*s < 0.001). The decrease in the average distance between individuals at the start indicated an increase in group cohesion, while the contrary was true for after the noise exposure. However, no significant differences were detected between treatments and there was also no treatment × period interaction. When each treatment was analyzed separately, all treatments had a significant effect on the group cohesion of the fish (repeated measures ANOVAs: *F*_(3,24)_ = 5.483, *P* = 0.005; *F*_(3,24)_ = 12.314, *P* < 0.001; *F*_(1.9,19.4)_ = 6.045, *P* = 0.010; *F*_(3,27)_ = 6.291, *P* = 0.002 for the continuous, regular 1-1, regular 1-9 and irregular treatment respectively). However, Bonferroni-corrected *post hoc* tests only showed a significant effect in the regular 1-1 treatment, where the distance between individuals in the first five-minute period of exposure was significantly lower than in the other periods (all *P*s < 0.05; Figure 3B). The effect only lasted for 4–5 min before the fish habituated to the noise and group cohesion resumed to the original level again.

Secondly, we also observed a significant increase in swimming speed at the start of the noise exposure, when all treatments were analyzed together (repeated measures ANOVA: *F*_(2.6,92.2)_ = 13.123, *P* < 0.001; Bonferroni-corrected *post hoc*: “during1” compared to other three periods, all *P*s < 0.001). Again, the effects of treatment and treatment × period were not significant. When each treatment was analyzed separately, the continuous and regular 1-1 treatment showed a significant increase in swimming speed of 4–5 cms^−1^ at the start of noise exposure (repeated measures ANOVAs: *F*_(3,24)_ = 5.491, *P* = 0.005; *F*_(2.1,16.5)_ = 7.413, *P* = 0.005 respectively). In addition, the irregular treatment showed a non-significant trend of an increase of 2–3 cms^−1^ (repeated measures ANOVAs: *F*_(2.0,18.2)_ = 2.697, *P* = 0.094). However, Bonferroni-corrected *post hoc* tests only revealed a significant effect in the continuous treatment (“before” compared to “during1”, *P* = 0.022). Figure 3A shows that the effect only lasted for 1 min.

Thirdly, when the treatments were analyzed together, we also observed a significant effect in the time that the fish spent in the top layer of the fish tank across periods (repeated measures ANOVA: *F*_(2.7,93.1)_ = 5.604, *P* = 0.002). Bonferroni-corrected *post hoc* tests showed that the fish spent significantly more time close to the surface at the start and right after the noise exposure compared to the last 5 min of the exposure (“during1” compared to “during2”, *P* = 0.017; “after” compared to “during2”, *P* = 0.006). There were no significant differences between treatments or a treatment × period interaction. When each treatment was analyzed separately, the regular 1-1 and irregular treatments both showed a significant effect (repeated measures ANOVAs: *F*_(3,24)_ = 3.800, *P* = 0.042; *F*_(3,27)_ = 4.183, *P* = 0.015 respectively), but Bonferroni-corrected *post hoc* tests only revealed a significant effect in the regular 1-1 treatment (“during1” compared to “during2”, *P* = 0.024).

### Experiment II (auditory preference test)

**Figure 4:**
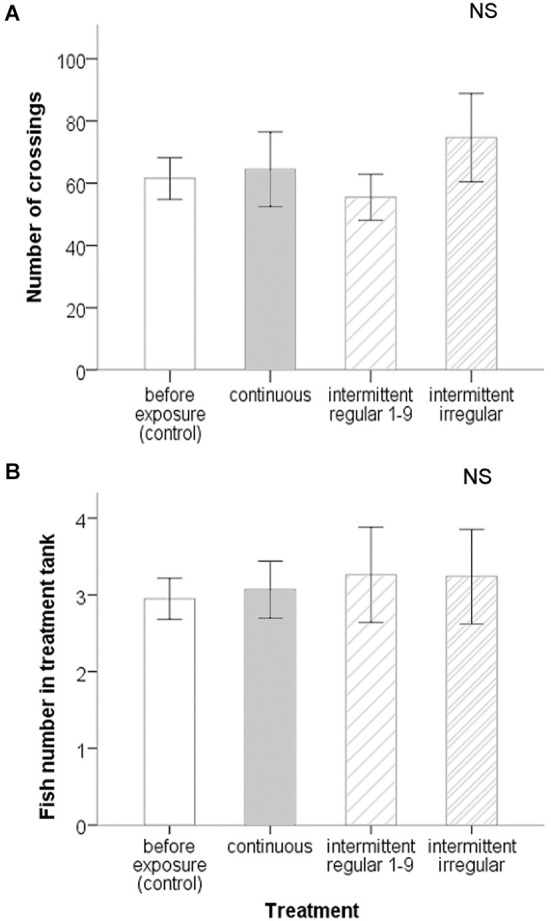
**Spatial response measures on groups of six zebrafish in the acoustic preference test of experiment II: (A) Number of crossings and (B) fish number in treatment tank (±SE) before treatments and during different treatments**. There were no significant differences before and during noise exposure. There were also no significant differences between treatments.

The six zebrafish individuals typically swam alone, in pairs or triplets, like in the first experiment, and also switched regularly between tanks. They swam readily across the tube tunnel between the two tanks, with an average of 62.56 ± 36.94 (S.D.) crossings per hour for a group of six fish before the treatments (Figure 4A). At the on-set of each noise exposure (sudden transition from 89 to 120–140 dB re 1 μPa), zebrafish in the noisy tank often showed distinct startle responses and often dived down to the bottom with fast and erratic swimming movements. Zebrafish that were in the quiet tank during the on-set did not show any startle response and were never observed diving down. All fish returned to regular swimming patterns within a few minutes while still exposed to noise.

The frequency of crossings between the quiet and noisy tank remained at levels that were similar to the pre-exposure exchange rate and did not change significantly in any of the treatments (ANOVA: *F*_(2,14)_ = 2.538, *P* = 0.115). Using also the repeated measures test, we found again no effect of period or treatment for the presence of fish in the noisy tank (ANOVA: *F*_(2,14)_ = 0.604, *P* = 0.560 and* F*_(2,14)_ = 0.342, *P* = 0.716 respectively), but we did find a period × treatment interaction (ANOVA: *F*_(4,28)_ = 2.846, *P* = 0.043), which can be attributed to a deviation from 3 fish present on each side before exposure for just the intermittent regular treatment. Taking all trials together, there were three fish in both tanks on average before noise exposure, as expected based on random distribution (Figure 4B). If we use the grouped value for the pre-exposure period (to avoid the effect of a Type I error), the number of fish in each tank remained the same and did not change significantly during noise exposure in any of the treatments (ANOVA: *F*_(3,47)_ = 0.143, *P* = 0.934).

## Discussion

Our study concerns the exploration of sound exposure effects on the behavior of captive zebrafish in two different experiments yielding two main results. The first experiment confirmed that sound can alter basic patterns of swimming behavior by changing group cohesion, swimming speed and swimming height. This effect occurred already at relatively low exposure levels and for all noise treatments. Intermittent stimuli some times appeared to induce stronger effects compared to continuous stimuli (differences in behavioral measures extending to more one-minute samples after exposure on-set), but responses were brief and variable and we did not find significant treatment effects. The second experiment revealed an unexpected lack of impact on spatial behavior despite relatively large differences in sound levels. Fish continued crossing between the two tanks at the same rate before and during the exposure, independent of the treatment. Despite an initially strong response to the sound exposure on-set, there was no evidence for a preference to be in the quiet fish tank over the noisy fish tank.

### Interpretation of sound-induced behavioral changes

In the first experiment, we conducted an acoustic exposure test without escape possibility, using a replicate series of forty groups of five zebrafish. We found several behavioral changes that can be compared to response patterns reported in other studies. The increase in group cohesion as observed at the onset of sound playback seems for example to match with an anxiety-indicating response to potential danger (Pitcher and Parrish, 1992; Speedie and Gerlai, 2008; Gerlai, 2010). This interpretation is in line with the observed increase in swimming speed, as hyperactivity has also been associated with perceived predation risk (Eaton et al., 1977; Rehnberg and Smith, 1988) and general anxiety (Cachat et al., 2010; Maximino et al., 2010; Stewart et al., 2012). In a study that also used groups of five zebrafish, test animals clearly formed tighter schools in response to alarm substance administered to the water (Speedie and Gerlai, 2008). In another study, groups of 16 zebrafish responded to a simulated predator flying overhead, first by a rapid decrease in group cohesion (swimming away from each other), followed secondarily by a steady increase in group cohesion (ending up closer to each other than in baseline conditions) (Miller and Gerlai, 2007).

However, an alternative explanation may even match better with the overall pattern of behavioral changes to the noise exposure in our first experiment. Shoaling intensity is typically not only determined by perceived danger of predator risk but can also be driven by foraging strategies and group-size related feeding opportunities (Ranta and Kaitala, 1991; Hoare et al., 2004). The increase in group cohesion as observed in our groups of five captive zebrafish was associated with increased swimming speed, but not with diving to the bottom, as would be expected from an anxiety-driven response (c.f. Sarà et al., 2007; Cachat et al., 2010). We even observed an increased tendency to approach the surface layer instead and individual fish often approached one another without staying in close proximity for long. Luca and Gerlai (2012) exposed individual zebrafish to a series of stimuli on a computer screen: three of these, a moving bird or an expanding dot from above and a sympatric predator fish on the side, clearly induced anxiety as reflected in erratic swimming, but also in a consistent downward shift to the bottom. We therefore speculate that the response behavior in our test may not reflect anxiety, but may be better explained by exploration for the potential presence of food. Our captive fish are used to get food at the surface, typically a little while after the animal care-taker would have entered the room, which may be associated with specific sounds that enter the fish tanks. Miller and Gerlai (2007) tested the impact of food on groups of 16 zebrafish and found a decreasing impact on group cohesion, although in that test food was actually available and visibly floating, spread-out, at the surface.

The exploration-for-food interpretation for the first experiment requires further investigation, but social influences on decision-making and foraging strategies are well-known and taxonomically widespread (van den Bos et al., 2013). Speeding towards other conspecifics in the upper layer of the fish tank could therefore well reflect social behavior that would improve chances of finding food, also referred to as “local enhancement”. It is also relevant to note in this context that sound is also known to play a role in food-finding under natural conditions. Historical studies on sharks (Family Carcharhinidae**)** reported that intermittent sounds are more of an attractant than continuous sounds because they resemble natural hydrodynamic sound patterns generated by potential prey items (Myrberg et al., 1969; Nelson and Johnson, 1972). In a more recent study, Holt and Johnston (2011) showed that cyprinid fish were attracted to the sound of rock shuffling, which may also concern a learned association with the potential for food items becoming available from under the rock. We believe that sound-induced aggregation as a potential foraging strategy deserves further study as it may benefit individual food intake and maybe even improve detection and localization of foraging opportunities through collective auditory sensitivity (c.f. Couzin, 2009; Webster and Laland, 2012).

### Lack of noise impact on spatial preference

In the second experiment, we conducted an acoustic preference test using twenty-four new groups of six zebrafish. We did not reveal a noise impact on the spatial distribution of zebrafish between the two tanks. The results showed that, when given an escape possibility, the zebrafish did not prefer the quiet tank over the noisy one. This finding was unexpected, especially considering the large SPL difference between the two tanks (>30 dB re 1 μPa) and the strong startle responses in the noisy tank at sound on-set, after which the fish often dived to the bottom with erratic swimming movements. This behavior indicates that the exposure condition of this second test at least initially led to some anxiety (c.f. Sarà et al., 2007; Cachat et al., 2010). However, the rapid return to regular swimming patterns and the absence of a tank preference suggests that the fish habituated quickly to the noise.

Although our results do not indicate a negative impact of noise exposure on captive zebrafish that extends beyond a few minutes, we are aware that we can not fully exclude an inability to respond spatially to the variation in acoustic conditions in our set-up. We can also not exclude yet physiological or more long-term consequences of the noisy test conditions (c.f. Wysocki et al., 2006; Purser and Radford, 2011). Furthermore, the specific social conditions of the loosely shoaling individual fish may have had an effect on the results of both experiments that may be worthwhile to investigate more (van den Bos et al., 2013). Guppies (*Poecilia reticulata*) were for example shown to be more likely to swim through a hole if that was observed before to be used by conspecifics (Laland and Williams, 1997). However, although social following tendencies, as well as socially modulated anxiety, may have played a role in decision-making and therefore in the absolute exchange rates of the acoustic preference test, they do not undermine the validity of our test and they do not explain a lack of noise-dependent spatial avoidance.

## Conclusions

The current findings reveal a clear impact of sound on the behavior of captive zebrafish, although there is no indication of potentially detrimental effects. Behavioral changes in the acoustic exposure test were brief and not straightforward indicators of anxiety. Nevertheless, the results do confirm that sound can be an important factor in captive fish studies that use behavioral read-outs, for example in pharmacological screening, especially if tests are brief and if laboratory tank systems are not well insulated acoustically. The lack of a spatial impact in the acoustic preference test, despite clear anxiety-related response patterns at the on-set, may indicate a lack of ability to avoid sound or a rapid habituation to the novel acoustic conditions. Also this result may suggest that sound does not form an immediate threat to fish in aquaculture or hobby or public aquaria, although we believe more studies are needed with different species, sound levels, and temporal patterns. Data are required especially for effects of repeated but unpredictable and uncontrollable sounds on behavioral changes, but also on the potentially underlying physiological changes (see e.g., Joëls and Baram, 2009; Lyons et al., 2009; Koolhaas et al., 2011; Steenbergen et al., 2011).

## Conflict of interest statement

The authors declare that the research was conducted in the absence of any commercial or financial relationships that could be construed as a potential conflict of interest.

## Footnotes

^1^http://www.random.org/
